# Prospects on Repurposing a Live Attenuated Vaccine for the Control of Unrelated Infections

**DOI:** 10.3389/fimmu.2022.877845

**Published:** 2022-05-16

**Authors:** Sang-Uk Seo, Baik-Lin Seong

**Affiliations:** ^1^ Department of Microbiology, College of Medicine, The Catholic University of Korea, Seoul, South Korea; ^2^ Department of Microbiology, Yonsei University College of Medicine, Seoul, South Korea; ^3^ Vaccine Innovative Technology ALliance (VITAL)-Korea, Yonsei University, Seoul, South Korea

**Keywords:** live attenuated vaccine, non-specific effects of vaccination, pandemic, genetic interference, innate immunity, trained immunity, heterologous immunity

## Abstract

Live vaccines use attenuated microbes to acquire immunity against pathogens in a safe way. As live attenuated vaccines (LAVs) still maintain infectivity, the vaccination stimulates diverse immune responses by mimicking natural infection. Induction of pathogen-specific antibodies or cell-mediated cytotoxicity provides means of specific protection, but LAV can also elicit unintended off-target effects, termed non-specific effects. Such mechanisms as short-lived genetic interference and non-specific innate immune response or long-lasting trained immunity and heterologous immunity allow LAVs to develop resistance to subsequent microbial infections. Based on their safety and potential for interference, LAVs may be considered as an alternative for immediate mitigation and control of unexpected pandemic outbreaks before pathogen-specific therapeutic and prophylactic measures are deployed.

## 1 Introduction

As members of the ecosystem, humans encounter an enormous number of microorganisms during their lifetimes. Microorganisms including bacteria, viruses, and parasites are present in the surrounding environment and within the human body. Many microorganisms, especially symbiotic bacteria which contribute to food digestion and immune development, are beneficial but a small fraction, which are termed ‘pathogens,’ can cause infectious disease (1). Infectious disease is the world’s leading cause of death, especially in low-income countries, but vaccines against pathogens effectively contribute to minimizing their impact (2). However, there are no vaccines against many dangerous pathogens. The ongoing coronavirus disease 2019 (COVID-19) outbreak caused by severe acute respiratory syndrome coronavirus 2 (SARS-CoV-2) led to excess mortality in the early stages of the outbreak due to the absence of effective vaccines. The experience with the COVID-19 pandemic points to the need for innovative vaccine platforms that can be adapted quickly upon the emergence of new pathogenic outbreaks.

Vaccine induces antigen-specific immune responses to a certain pathogen by exposing the pathogen’s antigen to an immune system in a safe manner. Although the responses vary by type of vaccine, vaccination can also provide partial protection against phylogenetically close variants due to the similarity of antigenic structures. However, antibody and cell-mediated responses induced by vaccination generally have no reactivity to unrelated pathogens. Moreover, vaccination is recommended ahead of pathogen circulation because adaptive immune response takes time to develop.

Besides the highly selective immunologic nature of vaccines, many studies report beneficial non-specific effects (NSEs) against off-target diseases (3). Unlike traditional vaccine effects, NSEs can be elicited immediately after vaccination and sometimes are perpetuated for longer periods. Live attenuated vaccine (LAV)-induced NSEs have been beneficial to non-infectious diseases such as type 1 diabetes, multiple sclerosis, and cancer (4), but in this review, we focus on the NSEs related to infectious diseases. Also, the review covers the basic concept of superinfection and interference that are important for understanding LAV-induced NSEs.

## 2 Superinfection

In a microorganism-enriched environment, human infection can involve multiple exogenous pathogens at the same time. Superinfection and coinfection are interchangeable terms that indicate multiple infections in a single host (5). These terms are generally used for pathogenic infections, which are distinct from harmless colonization by indigenous commensal bacteria. The latter do not produce symptoms and are seldom noticeable.

Many superinfections are known to synergistically augment disease severity. Respiratory viral infection by members of *Orthomyxoviridae*, *Paramyxoviridae*, and *Coronaviridae* are frequently associated with secondary bacterial infection (6, 7). Post-influenza secondary bacterial infection, especially with *Streptococcus pneumoniae*, is clinically important as this superinfection is commonly found in fatal cases (8, 9). Similarly, COVID-19-associated bacterial pneumonia, mostly associated with *Klebsiella pneumoniae* and *Staphylococcus aureus*, increase morbidity and mortality (10, 11). Primary infection with respiratory virus stimulates and makes changes in the immune system. Consequently, altered immunity sometimes adversely affects a secondary infection. For example, influenza A virus (IAV) infection induces robust interferon (IFN)-γ secretion and suppresses phagocytosis of alveolar macrophages which are key players in bacterial clearance (12). Similarly, type I IFNs (IFN-I) are important antiviral cytokines but IFN-I produced after IAV infection drives a hyporesponsive immune milieu during secondary bacterial infection (13). Immune responses against mixed infection are difficult to understand because there are so many variables in the course of superinfection. Further studies of the host’s response in a time-dependent manner using animal models are required as the immune response to primary infection constantly changes as the infection progresses. For example, in a mouse model, post-influenza pneumococcal pneumonia has increased susceptibility that doesn’t peak until about 7 days after IAV infection (14).

Secondary bacterial pneumonia, a well-known superinfection, results from serial infections in the lung. It seems natural that pathogenic synergism maximizes when superinfection occurs within the same organ, but in certain superinfections, two infections in different tissues can also be synergistic and aggravate the overall disease. This is common in chronic viral infections. For instance, chronic hepatitis C virus infection is associated with elevated risk for *Mycobacterium tuberculosis*, *S. aureus*, and *S. pneumoniae* infections (15–17). Human immunodeficiency virus (HIV) dramatically affects secondary bacterial infections due to disrupted cellular immunity. The HIV infection studies underscore the alteration of the immune system, an important factor that affects an individual’s susceptibility to superinfection.

## 3 Interference

### 3.1 Homologous Interference

Unlike the prevalent synergistic pathology discussed above, pathogens may form an antagonistic relationship, called ‘interference.’ Interference studies have been conducted using defective interfering (DI) virus particles that cannot replicate as they are missing essential genes (18). DI particles were recognized as having greater clinical importance in recent years when they were found to have interfering potential *in vivo* (19–21). Early studies also focused on the competition between two replication-competent viruses, especially between mutant and their parental wild-type (wt) viruses (22). As mutants largely maintain the original genetic sequences, these viruses can efficiently coinfect the same cell with wt virus and compete for the host’s mechanisms required for their replication. In general, mutant viruses generated by spontaneous or artificial mutation showed superior growth compared to parental wt virus during coinfection (22). The interference was observed in other RNA viruses including poliovirus (22, 23). These studies suggest that interference occurs at earlier steps of viral RNA synthesis. Interference was also reported in DNA viruses, including the temperature-sensitive herpes simplex virus type 1 (HSV-1) mutant that showed interference against a wt strain by delaying viral genome synthesis (24).

The early pioneering studies contributed to establishing the concept of viral interference. Homologous viral interference can be considered ‘genetic interference’ because proliferation of virulent strains is hindered by introduction of suboptimal mutant genomes. Unfortunately, interference at the genetic level based on an *in vitro* cell culture system has limitations for human disease studies because it is difficult to assess immune-mediated interference.

### 3.2 Heterologous Interference

Living multicellular organisms provide a niche for growth of both intracellular and extracellular microorganisms. For this reason, heterologous interference is common in humans and animals. Even harmless microorganisms compete with each other for energy sources and sometimes provide ‘colonization resistance’ against pathogenic infection (25, 26). Heterologous interference in living organisms is complicated because they induce counteracting host factors related to innate and adaptive immunity. Despite its complexity, study of heterologous interference is important to understand the pathophysiology of superinfection by unrelated pathogens.

IAV and influenza B virus (IBV) are antigenically distinct but both have significant clinical importance (27). IAV and IBV, like other RNA viruses, frequently yield variant strains that have mutations in antigenic sites. Antibodies against IAV or IBV may cross-react to homologous virus types. For example, IBV hemagglutinin-specific antibodies can provide protection against multiple IBV lineages (28). In contrast, IAV-specific antibodies induced by vaccination are not reactive to heterotypic (i.e., IBV) antigens (29). Thus, antibody-mediated immune response to IAV is not protective against IBV and *vice versa*. However, attenuated IAV infection provided shot-term protection against IBV in a mouse model (29). Moreover, IAV infection induced protection against IBV in the ferret model and it was achieved within short intervals of less than 7 days (30). Observed immediate protection was associated with decreased lung viral burden of secondarily infected virus. As antigen-specific adaptive immunity takes at least a week to reach the peak level, heterotypic interference should employ other mechanisms, such as innate immunity, to mitigate secondary viral propagation. Another study showed that surviving club cells, a type of lung epithelial cell that secret anti-inflammatory protein (31), are reprogrammed after IAV infection to elicit elevated antiviral responses (32). This antigenically unrelated interference between IAV and IBV was temporary, and animals eventually became susceptible again.

There have been other efforts to prove that IAV infection can provoke non-specific protection against unrelated pathogens. IAV-infected ferrets showed reduced viral burden after secondary respiratory syncytial virus (RSV) infection (33). When 3-, 7-, and 11-day intervals were tested, IAV infection suppressed RSV better during shorter intervals. The heterologous interference was not mediated by adaptive immunity because cross-reactive cellular response was minimal between IAV and RSV. In a mouse model, immediate protection (up to 7~14 days) against RSV was achieved by attenuated infection with IAV. Another study with IAV infection showed interference against the RSV challenge for a longer period (34). In that study, mice were challenged with RSV 5 weeks after IAV infection and showed reduced weight loss and histopathology. As in the ferret model, IAV-infected mice did not develop RSV-specific cellular immunity but transfer of splenocytes from IAV-infected mice resulted in reduced pathology after RSV infection. It was proposed that IAV-specific CD8^+^ T cells are recruited to RSV-infected lungs to exert bystander activity. This type of protection, called ‘heterologous immunity,’ will be discussed later in this review.

Systemic superinfection of lymphocytic choriomeningitis virus (LCMV) and ectromelia virus (ECTV) in mice also generated heterologous interference (35). When mice were infected with LCMV 1 or 2 days prior to ECTV infection, the coinfection extended survival of mice in an IFN-I–dependent manner; however, infection of LCMV after ECTV infection did not interfere with ECTV infection, indicating that timing of LCMV administration affects the degree of interference. In this case, the CD8^+^ T cell count in the spleen was comparable between coinfected and ECTV-only infected mice, suggesting immediate heterologous interference was mediated by innate immunity, such as IFN-I, rather than by long-lasting heterologous immunity.

Chronic infection of murine gammaherpesvirus 68 (MHV68) is an animal model for human gammaherpesviruses. MHV68-infected mice showed significantly lower mortality after lethal IAV infection compared to mock-infected control mice (36). The interference lasted for 60 days after the MHV68 infection but had disappeared by day 120. Adoptive transfer of alveolar macrophages isolated from MHV68-infected mice protected recipient mice from IAV challenge. In addition, MHV68 infection induced protection against another virus, bacteria, and malaria infections (37–39). Broad-spectrum heterologous interference achieved by MHV68 further suggests that protection is mediated by non-specific immune responses rather than by antigen-specific immune responses.

## 4 LAV-Induced NSE

LAV is a conventional prophylactic approach that has long been used against bacterial infections by *Mycobacterium* and *Salmonella*, and viral infections by polio, measles, mumps, rubella, varicella zoster, rota, yellow fever, smallpox, and influenza viruses. As the LAV is an infectious agent, people who are vaccinated could be at risk of superinfection, and at the same time be protected from secondary infection by interference (**Table 1**). Live attenuated influenza vaccine (LAIV) has been used against swine influenza, and superinfection of LAIV and a wt strain was detected in a vaccinated swine population (57). It is likely that humans administered the LAV often experience superinfection until LAV strains are cleared by host’s immune system. Also, human studies of Bacillus Calmette–Guérin (BCG) vaccine suggest that LAVs can elicit beneficial protective effects on subsequent infection by unrelated pathogens (41).

**Table 1:** Beneficial non-specific effects against unrelated pathogens induced by live attenuated vaccine (LAV) administration *in vivo*.

LAV	Target pathogen	Study model	Vaccination route	Interval*	Outcome of vaccination	Suggested mechanism	Reference
BCG	ECTV	Mouse	IP	3 weeks	Reduced viral burden	Enhanced of interferon response	(40)
	EMCV	Mouse	IP, IV	5 weeks	Increased survival rate	Macrophage-mediated innate immunity	(41)
	HSV-1	Mouse	IV	21-49 days	Increased survival rate	Stimulation of phagocytes	(41)
	HSV-2	Mouse	IV	15-31 days	Increased survival rate	Stimulation of phagocytes	(41)
	HSV-2	Mouse	ID, IP	6 days	Increased survival rate	ND	(41)
	HPV	Human	Topical application	NA	Complete or partial resolution of warts	ND	(41)
	HPV	Human	ID	NA	Complete or partial resolution of warts	ND	(41)
	IAV	Human	ID	NA	Increased and accelerated IAV-specific antibody response	ND	(41)
	IAV	Mouse	IN, IP	4-12 weeks	Increased survival rate	ND	(41)
	IAV	Mouse	IN, SC	2 days	IN BCG vaccination increased survival rate	Enhanced efferocytic ability of alveolar phagocytes	(41)
	IAV	Mouse	IV, IN	14-31 days	IV BCG vaccination increased survival rate	Stimulation of phagocytes	(41)
	IAV	Mouse, hamster	IV	4-6 weeks	Increased survival rate, reduced weight loss	Trained immunity	(42)
	JEV	Mouse	SC	15 days	Delayed onset of clinical symptoms and death	Anti-inflammatory effect	(41)
	Malaria	Human	ID	5 weeks	Decreased parasitemia	Trained immunity	(43)
	RSV	Human	ID	NA	Reduced risk of acute lower respiratory tract infection	ND	(41)
	SARS-CoV-2	Human	ID	NA	Decreased anti-SARS-CoV-2 seroprevalence	ND	(44)
	SARS-CoV-2	Human	ID	NA	Lower mortality rate	ND	(45)
	SARS-CoV-2	Human	ID	NA	Reduced incidence of new infection	Epigenetic reprogramming and increase cytokine production	(46)
	SARS-CoV-2	Mouse	SC	3 weeks	Reduced weight loss	ND	(47)
	SARS-CoV-2	Mouse	IV	6-16 days	Increased survival rate, reduced weight loss	Nonspecific stimulation of the pulmonary immune response	(48)
	Vaccinia virus	Mouse	SC	> 4 weeks	Reduced viral burden	T cell-mediated heterologous immunity	(49)
	Yellow fever virus	Human	ID	4 weeks	Reduced viral burden	Epigenetic reprogramming in monocytes	(50)
*Bordetella pertussis*	IAV	Mouse	IN	3-12 weeks	Increased survival rate, reduced histopathology	Anti-inflammatory effect	(51)
	RSV	Mouse	IN	7-9 weeks	Reduced viral burden	Enhanced IL-17 response and immune cell recruitment	(52)
LAIV	IBV	Mouse	IN	0-4 days	Increased survival rate	Enhanced pro-inflammatory cytokine and interferon responses	(29)
	RSV	Mouse	IN	2-28 days	Reduced viral burden	Enhanced pro-inflammatory cytokine and interferon responses	(53)
	SARS-CoV-2	Ferret	IN	0-3 days	Reduced viral burden	ND	(54)
OPV	SARS-CoV-2	Human	Oral	NA	Reduced symptomatic infection	ND	(55)
*Salmonella*	IAV	Mouse	IN, Oral	1 day	Increased survival rate	Stimulation of innate immune responses	(56)

*Interval between vaccination and experimental challenge.

BCG, Bacillus Calmette-Guérin; ECTV, ectromelia virus; EMCV, encephalomyocarditis virus; HPV, human papillomavirus; HSV, herpes simplex virus; IAV, influenza A virus; IBV, influenza B virus; ID, intradermal; IN, intranasal; IP, intraperitoneal; IV, intravenous; JEV, Japanese encephalitis virus; LAIV, live attenuated influenza vaccine; NA, not applicable; ND, not determined; OPV, oral poliovirus vaccine; RSV, respiratory syncytial virus; SC, subcutaneous; SARS-CoV-2, severe acute respiratory syndrome coronavirus 2.

### 4.1 Bacterial LAVs

BCG vaccine is an attenuated *Mycobacterium bovis* strain developed to protect against tuberculosis. BCG is known for its interference potential against viral infections, including ECTV, encephalomyocarditis virus, HSV-1, HSV-2, human papillomavirus, IAV, Japanese encephalitis virus, RSV, and vaccinia virus (41). Epidemiological studies have shown that all-cause mortality, including non-tuberculous infections, is reduced by BCG vaccination in early life (58–60). BCG also has antifungal effects against *Candida albicans* and *Cryptococcus neoformans* (61, 62). Most of the antifungal effects were assessed using immune-disrupted SCID or IFN-γ knock-out mice. Thus, further validation is required.

Similarly, interference against influenza infection by attenuated bacteria is achieved by oral and nasal administration of *Salmonella* LAV (56). As *Salmonella* LAV has been widely studied as a mucosal vaccine platform that expresses foreign antigen and is usually introduced orally (14), the potential of interference against infections in different mucosal sites need further study. In other attempts for LAV development, live attenuated *Bordetella pertussis* vaccine showed protection against influenza and RSV infections (51, 52). Of note, most interference induced by bacterial LAVs was against viral infections.

### 4.2 Viral LAVs

#### 4.2.1 LAIV

A reverse genetic approach has been frequently used to generate LAIV strains (63, 64), but currently licensed LAIVs were generated by cold-adaptation. Three individual cold-adapted (ca) LAIV backbone strains were prepared by passaging virulent strains at progressively lower temperatures (65–67). These ca backbone strains were used to generate a vaccine strain that contains surface glycoprotein gene segments of circulating virus. For example, hemagglutinin (HA) and neuraminidase (NA) genes of the ca-backbone strain, A/Ann Arbor/6/60 ca (H2N2), were replaced by those of A/Korea/1/82 wt (H3N2) to yield the A/Korea/1/82 ca vaccine strain. As many temperature-sensitive mutant viruses showed homologous interference against parental wt viruses, A/Korea/1/82 ca genes were dominantly synthesized compared with wt genes when these two viruses coinfected MDCK cells (68). The interference of ca LAIV *in vivo* was confirmed by mixed infection of ferrets (69). Ferrets coinfected with wt and ca vaccine strains produced antibodies to both strains, indicating these two viruses were propagated. While wt virus-infected ferrets showed fever and coryza for 3 days, those symptoms were not observed in wt and ca coinfected ferrets. Interestingly, the interference by ca vaccine (H3N2) was effective for both homosubtypic (H3N2) and heterosubtypic (H1N1) wt challenge. In a double-blind human coinfection study, volunteers who received both wt and ca virus had lower symptom scores than those given only wt virus, although the difference was not statistically significant (70). The series of studies based on A/Ann Arbor/6/60 ca strain extended the use of LAIV from conventional homologous protection to a broader range of influenza virus infections.

#### 4.2.2 Other Viral LAVs

Based on recommended immunization schedules, children receive various vaccines against viral pathogens early in life. Measles vaccine has long been used in low-income countries and children get their first vaccinations at age 9 months. Analysis of 10 cohort studies showed 30% to 85% protective efficacy against all-cause mortality in developing countries (71). NSEs on mortality were particularly effective in children who did not receive neonatal vitamin A (72). Another randomized trial showed that measles vaccine protects against hospital admissions, indicating that NSEs reduce both morbidity and mortality in low-income countries (73). A lower risk of hospital admission was also reported in Danish children vaccinated with measles-mumps-rubella (MMR) vaccine (74). These findings indicate that NSEs conferred by measles vaccine are beneficial regardless of an area’s economic status.

Live enterovirus vaccines (LEVs), including oral polio vaccine (OPV), use nonpathogenic viruses to prevent various enterovirus diseases. While the LEVs successfully control pathogenic enterovirus infection as intended, they also exert prophylactic effects against seasonal influenza (75). OPV is currently recommended for administration at birth and thus can produce NSEs even earlier than measles vaccine. In a randomized trial, OPV showed protection against infant mortality associated with infectious diseases (76). As with MMR vaccinations, OPV was also associated with a lower risk of hospitalization in Denmark (77).

Live attenuated rotavirus vaccine has been proposed as a mechanism to protect against non-rotavirus gastroenteritis (78). However, this concept needs further evaluation because of a conflicting report (79). Overall, accumulated evidence suggests that other viral LAVs may also provide protection against unrelated pathogenic infections.

## 5 Mechanisms of LAV-Induced NSEs

**Figure 1:**
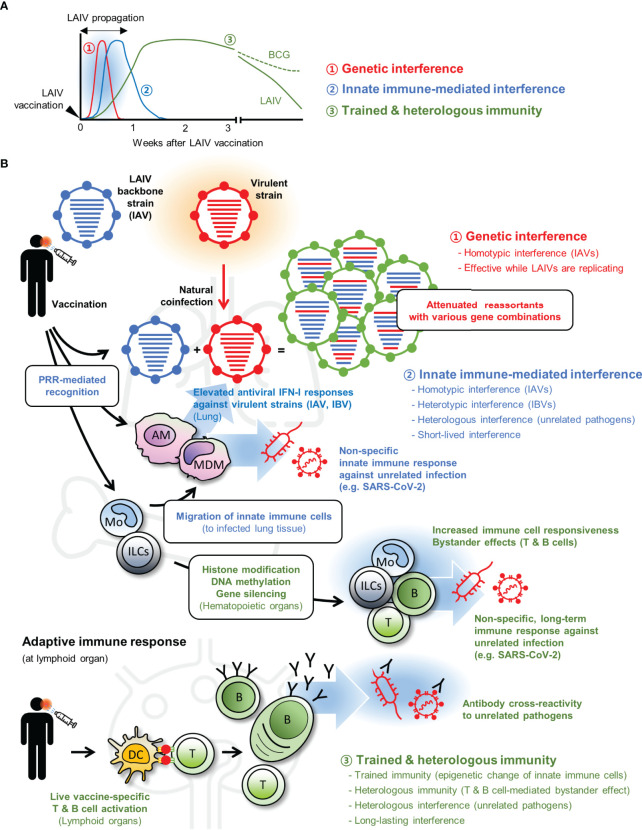
Live attenuated vaccine (LAV)-induced non-specific effects (NSEs). **(A)** LAIV can induce three different types of interference (1, genetic interference; 2, innate immune-mediated interference; and 3, trained and heterologous immunity) with each protective effect elicited at different time points after vaccination. **(B)** When virulent strain and LAIV are coinfected, both viruses can infect the same cell and produce progenies with diverse genomic combinations. Generation of suboptimal progenies induces ‘genetic interference’ during early infection (shown in red). At the same time, LAIV vaccination triggers innate immune-mediated interference (shown in blue) *via* stimulation of pattern recognition receptors (PRRs). Many immune cells, including alveolar macrophage (AM), monocyte (Mo), monocyte-derived macrophage (MDM), elicit antiviral type I interferon (IFN-I) response and provide broad-spectrum protection to homotypic influenza A virus (IAV), heterotypic influenza B virus (IBV), and other heterologous unrelated pathogens. Whereas the innate immune-mediated interference subsides after LAIV clearance, LAIV can induce long-lasting interference (shown in green). LAIV administration triggers epigenetic alteration in innate immune cells including innate lymphoid cells (ILCs) and Mo (trained immunity) and prolonged bystander effects by LAIV-specific T and B cells (heterologous immunity); both contribute to boost host resistance against unrelated pathogens in an antigen-independent manner. In some cases, LAIV-specific antibodies may cross-react with unrelated pathogens. Genetic interference is an LAIV-specific event, but innate immune-mediated interference and long-term interference can be induced by other LAVs. Certain LAVs, such as Bacillus Calmette–Guérin (BCG), can elicit longer trained and heterologous immunity. DC, dendritic cells.

Beneficial effects of LAV-induced interference suggest its potential use for the control of unrelated infectious diseases that lack efficient prophylactic and therapeutic measures. Although licensed LAVs are confirmed for their safety by clinical trials, new concerns may arise when vaccines are applied for different uses. Superinfection of LAVs and virulent pathogens is unavoidable for interference and may advertently enhance the pathology of secondary infections (80, 81). Therefore, to assure the safety of non-conventional LAV use, it is essential to determine the underlying mechanisms of vaccine-induced NSEs (**Figure 1**).

### 5.1 Genetic Interference

Genetic interference to based on the replication dominance of attenuated virus. For this reason, genetic interference by LAV is observed in interrelated pathogens. In addition to genetic dominance studies using A/Ann Arbor/6/60 ca LAIV, mechanistic analysis of genetic interference was performed with another LAIV backbone strain, X-31 ca (H3N2). When X-31 ca was administered to mice 1 to 4 days before virulent challenge with heterosubtypic A/New Caledonia/20/99 (H1N1), these mice showed reduced weight loss and mortality compared to non-vaccinated control mice (29). Even simultaneous introduction of X-31 ca with virulent virus showed protective effects. As this early protection was achieved without specific antibody responses, which started to increase 5 days after vaccination, interference was analyzed at the genetic level. IAV has eight segmented genomes and coinfection of two viruses enables them to interchange their genes to generate various combinations of reassortant progenies. Attenuated, temperature-sensitive, and ca phenotypes are the consequence of accumulated mutations dispersed in the segmented RNA genome (65, 82). Thus, an increased ratio of reassortant progenies that contain ca-originated gene(s) contributes toward overall attenuation of virulence. The ratio of reassortant virus was 10% to 46% in mice that received both wt and ca viruses at different intervals, indicating active genetic exchanges occur *in vivo*. Clearly, LAIV can maximize the genetic interference based on the advantage of segmented gene structure.

As shown in interference between virulent IAV and IBV (30), X-31 ca induced protection against heterotypic IBV challenge in mice (29). Basically, genetic interference can be best achieved when genes of two viruses are compatible. Although IBV contains segmented genomes with similarity in terms of protein functions they encode, natural reassortment between influenza A and B viruses has not yet been reported. However, the polymerase complex of IAV and IBV recognize the non-coding terminal sequence of heterotypic genes and the polymerase complex that contains heterotypic subunits are still functional albeit at a low level (83–85). Genetic interference seems clearly to contribute interference between homotypic IAV infections. Although the possibility of heterotypic genetic interference between IAV and IBV were suggested by other studies, it seems other mechanisms, including immediate induction of innate immune response, play a major role.

### 5.2 Innate Immunity

Innate immunity provides general barrier function because it is designed to respond to a wide range of microbial infections. Therefore, innate immunity induced by certain microorganisms can elicit broad protection against other pathogens. Vaccination with LAIV induces innate immunity similar to natural infection as the vaccine contains every element required for self-replication and actually ‘infects’ the host (86). Although LAIV administration is generally asymptomatic, it elicits sufficient innate immune response (87).

As a prototype LAIV, X-31 ca induced the production of multiple inflammatory cytokines and IFN-I (29, 53). Such inflammatory and IFN responses are initiated *via* pattern recognition receptors (PRRs). Toll-like receptor (TLR) 3 and 7 are major intracellular sensors for viral RNA genomes and recognize influenza virus infections (88, 89). Mice with defects in TLR signaling were highly susceptible to infection, confirming the crucial role of TLR-mediated innate immunity in protection against IAV (90). When TLR3 and 7 agonists were administered, they elicited immediate protection against IAV challenge as seen with LAIV vaccination (29). Other studies also proved that agonists for TLRs, including TLR2, 3, 4, and 7, induced therapeutic effects on influenza infection (91). When tested for prophylactic effects, TLR agonists protected mice against different strains including H5 avian influenza and 2009 pandemic strains within several days (92–96). Agonists targeting other PRRs, including RIG-I and NOD2, also induced antiviral innate immune responses and protected mice from IAV infection (97, 98). Based on extensive studies that proved the protective innate immune response *via* PRR stimulation, LAIVs are expected to be sufficiently immunogenic to mitigate subsequent viral infection, even antigenically unrelated pathogens. Indeed, when X-31 ca was administered 2 days before RSV challenge, it significantly suppressed RSV propagation in a TLR3- and TLR7-dependent manner (53).

IFN-I triggers the expression of multiple IFN-stimulated genes (ISGs) that cooperate to inhibit the virus replication cycle in infected cells (99). For example, IFN-induced transmembrane (IFITM) family proteins restrict entry (100), Mx GTPases block early replication of viral genomes (101), and 2’-5’-oligoadenylate synthetase (OAS) family proteins degrade viral genomes by activating the ribonuclease (RNase) L (102). Virus infection is the main stimulator that potentiates cells to produce IFN-I *via* PRR signaling (88, 103, 104). Likewise, LAIV induces IFN-I in the lung shortly after vaccination (29, 53). Even non-viral stimulation can increase the host’s antiviral status by inducing IFN-I production (105). IFN-I induced by host DNA in a mouse lung fibrosis model attenuated IAV infection (106). It is important to note that ISGs, which can be produced by LAIV and other LAVs, also induce antiviral effects to unrelated viral infections. Furthermore, IFN-I not only provides direct protection to infected cells but also increases the antiviral status of non-infected cells, which together mitigate overall virus propagation in infected hosts.

IFN-γ, another subtype of IFN, is involved in both innate and adaptive immune responses. Vaccination with BCG allows the host to induce prolonged IFN-γ responses that contribute non-specific protection against ECTV and vaccinia virus (40, 49).

### 5.3 Long-Lasting Immunity

#### 5.3.1 Trained Immunity

Vaccination with BCG maintains a prolonged NSE, and even acute IAV infection maintained NSEs over 1 month (34, 107). The immune mechanism that drives prolonged immune response by LAV seems to differ from those of immediate or short-lived interference. The concept of ‘trained immunity,’ also known as ‘innate immune memory,’ was established based on the observation that innate immune cells of recently challenged hosts retain the elevated responsiveness to subsequent microbial stimulation (108). When a host experiences an initial challenge, innate immune cells, including monocytes, macrophages, and natural killer cells, undergo epigenetic changes and this genetic reprogramming affects how immune cells respond to secondary infection. Bone marrow and spleen are hematopoietic organs where immune cells are differentiated and educated. BCG can directly access the bone marrow upon vaccination and induces epigenetic modification of hematopoietic stem cells (HSCs) in mice (42). These reprogrammed monocytes are protective against *M. tuberculosis* infection; however, heterologous protection against unrelated pathogens was not tested.

A human study showed that BCG administration trained monocytes to produce more pro-inflammatory cytokines upon unrelated microbial stimulation in a NOD2-dependent manner (61). Macrophages are an important source of immune modulators for pro-inflammatory and anti-inflammatory responses. As monocytes are recruited to the site of infection and can differentiate into monocyte-derived macrophages, alteration of the transcriptomic profile by epigenetic change will significantly affect the pathologic outcome. In consecutive human studies, nonspecific protection by BCG vaccination was proved by experimental infection with attenuated yellow fever virus or malaria (43, 50). Genome-wide epigenetic changes were observed in monocytes and early activation of innate immune cells, including neutrophils, NK cells, and monocytes was observed in BCG-vaccinated adult volunteers. These subjects developed earlier symptoms of malaria infection, but it eventually was associated with lower parasitemia (43). BCG vaccination did not alter the composition of immune cells and their progenitors but reprogrammed gene transcription to draw functional alteration in CD14^+^ peripheral monocytes (109). Trained immunity lasted more than 90 days. Thus, BCG vaccination may induce prolonged NSEs against pathogenic infections. Because these experimental human studies were in adults, trained immunity induced by early BCG vaccination needs further study.

Of note, BCG vaccination induces immediate interference. Neonatal BCG vaccination induced granulocyte colony-stimulating factor and activated emergency granulopoiesis (110). Elevated neutrophil numbers were associated with reduced mortality by polymicrobial sepsis in vaccinated neonates and the protection was achieved within 3 days. The study indicates that long-lasting NSEs can be achieved quickly after vaccination.

#### 5.3.2 Heterologous Immunity

Heterologous immunity is another term that is used to explain prolonged NSEs, but this phenomenon mainly focuses on lymphocyte responses rather than on innate immune cells. Heterologous immunity may have been induced based on cross-reactive T cell antigens between unrelated pathogens (111–113). While heterologous immunity can provide protection against experimental infections (111, 114), in some cases, heterologous immunity can adversely increase disease severity through cross-reactive CD8^+^ T cells. Two studies reported that the degree of acute infectious mononucleosis caused by Epstein-Barr virus infection correlated with development of IAV-specific cross-reactive memory T cells (115, 116). Therefore, heterologous immunity induced by LAVs can be beneficial but also can be detrimental during subsequent pathogenic infections through the activation of cross-reactive lymphocytes.

Alternatively, heterologous immunity can interfere with heterologous infection through the antigen-independent bystander effects of lymphocytes. When peripheral blood mononuclear cells were isolated from BCG-vaccinated individuals and stimulated *ex vivo* with unrelated microbial stimulants, increased production of Th1- and Th17-related cytokines were detected. When mice were infected LCMV, it generated LCMV-specific CD8^+^ T cells that produced IFN-γ in lungs and draining lymph nodes. Subsequent systemic vaccinia virus infection re-activated these cells to produce increased serum IFN-γ compared with mock-infected control mice (117). Influenza infection also induces similar long-lasting IFN-γ producing CD8^+^ T cells and suppresses Th2 responses induced by RSV infection (34). These results suggest that LAV administration, in general, boosts Th1 and Th17 responses in the early phase of secondary infection and contributes to attenuation of overall pathology by secondary infection.

Long-lasting NSEs are mediated by mixed contributions of innate and adaptive cell populations. Both mechanisms may induce cellular reprogramming in hematopoietic organs, such as bone marrow and spleen, after primary infection (118). Further studies are required to define the detailed mechanism of how LAV administration communicates with hematopoietic organs to educate immune cells.

## 6 Use of LAVs for Control of Newly Emerging Viruses

### 6.1 NSEs Induced by LAVs Against SARS-CoV-2

New viral infectious diseases, including highly pathogenic avian influenza, 2009 pandemic influenza, SARS, and Middle East respiratory syndrome, have evolved during the last two decades. More recently, COVID-19 spread rapidly and yielded more than 481 million confirmed cases as of 29 March 2022. SARS-CoV-2 vaccines were developed in an exceptionally short time period and applied to human use under provisional approval beginning December 2020. COVID-19 caused significant social and health disruptions worldwide in the early stages of the pandemic and options to minimize the disease burden were discussed.

Because of the well-known heterologous protection of BCG vaccination, its potential use for control of COVID-19 was suggested (119, 120). Multiple clinical studies have assessed the impact of BCG vaccination against COVID-19. These fall into two categories (1): the effect of childhood BCG vaccination on COVID-19 and (2) the effect of re-vaccination on COVID-19 in adults. BCG vaccination in childhood was associated with decreased SARS-CoV-2 prevalence or mortality among health care workers (44, 45); however, in clinical studies, the protective effect of re-vaccination with BCG in adults was controversial. One randomized clinical trial showed that BCG vaccination in the elderly was correlated with lower incidence of SARS-CoV-2 infection (46). Yet other studies found no significant protective effect with BCG vaccination (121–123). Similarly, studies of COVID-19 in animal models also produced contradictory results. In studies using mice expressing the human SARS-CoV-2 receptor, administration of BCG vaccine reduced morbidity and mortality upon SARS-CoV-2 infection (47, 48). But in studies with mice, hamsters, and rhesus macaques, BCG vaccination did not protect animals from SARS-CoV-2 infection (124, 125). The results need to be compared in terms of animals used, study methods and administration routes, and BCG strains. Despite the disparate findings, the positive results support the possible use of BCG vaccine for NSE-mediated control of the current pandemic. In addition, one study reported that peptides derived from BCG vaccine induced SARS-CoV-2-specific T cells based on antigen homology (126). That study showed that heterologous immunity can be induced by BCG vaccination.

OPV may also induce heterologous protection (119). Mothers who were passively exposed to OPV by their children had a decreased incidence of COVID-19 infection (55). This suggests that OPV exposure may be one means of lowering the viral load of unrelated viruses in a population. Another attempt to induce heterologous protection using DI poliovirus particles showed promising prophylactic and therapeutic effects against various RNA virus infections including SARS-CoV-2 (127). When mice received the defective poliovirus genome intranasally, DI particles were generated to induce systemic IFN-I–dependent antiviral responses. This successful example of IFN-I–mediated protection suggests LAV-mediated IFN-I production, which is also induced by LAIV, will similarly protect hosts from SARS-CoV-2 infection.

During the influenza season, there were concerns that people infected with COVID-19 might experience comorbidity. Although coinfection with SARS-CoV-2 and influenza did not affect the overall mortality rate, some studies suggested significant increases in morbidity and mortality (128, 129). In a mouse model, coinfection with SARS-CoV-2 and IAV resulted in increased mortality and severe histopathology (130). However, coinfection with SARS-CoV-2 and LAIV did not enhance the histological pathology in the ferret model (54). In the latter study, prior administration of LAIV 3 days before challenge reduced the replication of SARS-CoV-2 from throat swabs, indicating the potential of LAIV as an immediate prophylactic measure for highly pathogenic coronavirus infection.

### 6.2 Control of Unrelated Pathogens in Future Pandemics

BCG vaccine has been used for more than 100 years and administered to billions of children (131). As planned, BCG vaccination significantly lowered the incidence of tuberculosis while simultaneously providing unintended beneficial effects. The COVID-19 outbreak provided an opportunity to study the NSEs of BCG and other vaccines. Although the effectiveness of LAV-induced NSEs against SARS-CoV-2 needs further study, the evidence of BCG and OPV vaccination-induced interference suggests promising potential therapeutic use of LAV in future pandemics, especially during early stages when vaccines and drug therapies have not been developed.

**Figure 2:**
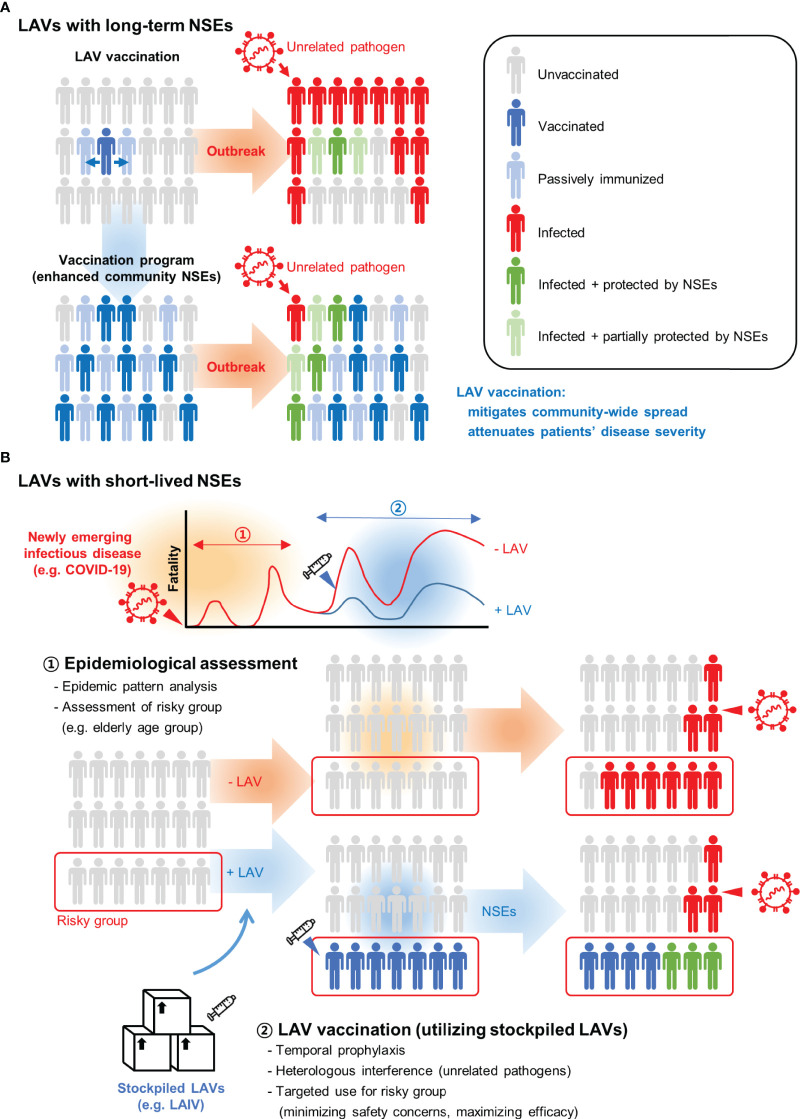
Strategic use of live attenuated vaccines (LAVs) for unrelated pathogen outbreaks. **(A)** LAVs that elicit beneficial non-specific effects (NSEs) can be transferred from a vaccinated person to passively immunized contacts. Persons exposed to LAV may develop NSEs that attenuate the infection of unrelated pathogens. With higher vaccination rates, community-wide NSEs mitigate the spread of pathogens and limit the impact of an outbreak. **(B)** LAVs with short-lived NSEs need to be administered during the outbreak. This approach can be effective in the early stage of newly emerging infectious diseases when no effective vaccines and drugs are available. The optimal timing of vaccination must be determined through epidemic pattern analysis. Also, for targeted vaccination, it is important to identify groups at highest risk for fatality. For example, most fatalities were elderly people during the COVID-19 outbreak. A minimal dose of live attenuated influenza vaccine (LAIV) can be selectively administered to an at-risk population to reduce the fatality rate. Stockpiled LAIVs such as pre-pandemic or seasonal vaccines can be considered for alternative use during unexpected new respiratory virus outbreaks.

Results of animal and human studies found that NSE duration differs by type of LAV (**Figure 2**). For example, BCG vaccines led to long-lasting NSEs while LAIVs typically were associated with short-lived effects. Therefore, two distinct types of NSE-based vaccine programs need to be considered. Long-lasting NSEs induced by BGC and OPV vaccination can be considered as passive outcomes. Epidemiological studies showed that effectiveness of vaccination correlates with population-wide coverage (132). Thus, NSE-based protection will be more effective in communities with higher LAV coverage (133). The beneficial effects associated with long-lasting NSEs will be most striking when sufficient vaccines are provided to countries with poor medical infrastructures (**Figure 2A**). Pending the distribution of vaccines, we suggest that LAIVs might be a solution for immediate protection against newly emerging infectious pathogens. However, this approach has been proven mostly at the research level and studies are urgently needed to address safety issues related to NSEs resulting from superinfection of the LAIV and the pathogen. There needs to be an assessment of the risk from superinfection and the expected benefits from NSEs. For example, before the availability of vaccines, most COVID-19 fatalities occurred in the elderly. Assuming this scenario is likely in future pandemics, it seems reasonable to selectively administer LAIV to the elderly in communities in which the infection is actively spreading. Another hurdle for immediate use of an NSE strategy is vaccine supply. To solve this problem, planning might focus on alternative uses of stockpiled pre-pandemic and seasonal LAIV (**Figure 2B**).

## 7 Conclusion

Although a strategic use of LAV-induced NSEs appears promising, the immediate concern is safety and effectiveness in humans. However, new infectious diseases will continue to emerge in the post-COVID-19 era for which no effective therapeutic or prophylactic means are available. Based on extensive research and clinical analysis, we suggest that the use of LAVs not only strengthens the preparedness for future pandemics but also provides a beneficial option for controlling infectious diseases in the absence of licensed vaccines. A balanced view of the pros and cons of use of LAVs must be developed as a basis for a judicious choice of LAV candidates for immediate mitigation and control of unexpected pandemic outbreaks.

## Author Contributions

S-US and B-LS contributed to the preparation of the manuscript. All authors contributed to the article and approved the submitted version.

## Funding

This work was supported by the Korea Health Technology R&D Project through the Korea Health Industry Development Institute (KHIDI), the Ministry of Health & Welfare (HV20C0070), and the Bio & Medical Technology Development Program of the National Research Foundation (NRF) funded by the Ministry of Science & ICT (2021M3E5E3080927).

## Conflict of Interest

The authors declare that the research was conducted in the absence of any commercial or financial relationships that could be construed as a potential conflict of interest.

## Publisher’s Note

All claims expressed in this article are solely those of the authors and do not necessarily represent those of their affiliated organizations, or those of the publisher, the editors and the reviewers. Any product that may be evaluated in this article, or claim that may be made by its manufacturer, is not guaranteed or endorsed by the publisher.
